# Green Preparation of Durian Rind-Based Cellulose Nanofiber and Its Application in Aerogel

**DOI:** 10.3390/molecules27196507

**Published:** 2022-10-02

**Authors:** Huwei Xing, Yongsheng Fei, Jingru Cheng, Congcong Wang, Jingjing Zhang, Chenxi Niu, Qian Fu, Jiali Cheng, Lingbin Lu

**Affiliations:** Special Glass Key Lab of Hainan Province, School of Materials Science and Engineering, Hainan University, Haikou 570228, China

**Keywords:** durian rind, cellulose nanofiber, dimethyl sulfoxide, ultrasonic, aerogel

## Abstract

In this study, a green, highly efficient and low energy consumption preparation method of cellulose nanofiber (CNF) was developed by using agricultural and forestry waste durian rinds as raw materials. The power of ultrasonic treatment was successfully reduced to only 360 W with low molecular weight liquid DMSO. The obtained durian rind-based CNF had a diameter of 8–20 nm and a length of several micrometers. It had good dispersion and stability in water, and could spontaneously cross-link to form hydrogel at room temperature when the concentration was more than 0.5%. The microscopic morphology and compressive properties of CNF aerogels and composite cellulose aerogels prepared from durian rind-based CNF were evaluated. It was found that CNF could effectively prevent the volume shrinkage of aerogel, and the concentration of CNF had a significant effect on the microstructure and mechanical properties of aerogel. The CNF aerogel with 1% CNF exhibited a sheet structure braced by fibers, which had the strongest compression performance. The porosity of CNF aerogels was high to 99%. The compressive strength of the composite cellulose aerogel with durian rind-based CNF was effectively enhanced.

## 1. Introduction

With the rapid development of modern industry and the excessive use of petroleum-based materials, the energy crisis is becoming more and more serious. The non-biodegradable and unsustainable characteristics of petroleum-based materials have also caused serious environmental pollution [1,2]. Therefore, the use of renewable resources to explore natural functional materials has received more and more attention. Cellulose is composed of D-glucose linked by β-1,4 glycosidic bonds, and is the most abundant natural polymer in nature [3]. It has attracted much attention due to good biocompatibility, biodegradability and sustainability. Cellulose comes from a wide variety of sources, such as wood, cotton, grass, flax, fungi, algae, etc., which have good sustainability [4,5]. Wood and cotton are the main sources of cellulose. However, due to the long growth cycle and shortage of wood, it is a limited natural resource from an environmental point of view. Cotton is mainly used in the textile industry and is not the best source of cellulose. Therefore, the extraction of cellulose from agricultural and forestry wastes has become a research hotspot in recent years. Known as ‘the king of fruits’, durian is a popular fruit in Southeast Asian countries such as Thailand, Indonesia, the Philippines and Malaysia. Durian has high nutritional value, medicinal value and economic value. Durian rinds are usually discarded as waste, which pollutes the environment. Durian rinds contain about 31–35% cellulose, 10–11% lignin and 15–18% hemicellulose (dry basis) [6]. The utilization of durian rinds can greatly improve the value of durian rinds and help to alleviate the energy crisis.

Cellulose nanofiber (CNF) is a kind of nanofiber produced from cellulose by high-energy mechanical homogenization. Its length reaches the micrometer scale, while its diameter is only 2–50 nm, with a high aspect ratio [7]. Due to its large specific surface area, high strength, dimensional anisotropy and natural origin [8], the functionalization of CNF has attracted extensive research interest. Extensive work has been carried out to obtain CNF from agricultural and forestry wastes. Morán et al. treated sisal fibers with 60% sulfuric acid to obtain CNF [9]. Sankhla et al. prepared bagasse-based CNF with ionic liquids [10]. Concentrated sulfuric acid is highly corrosive and high-cost for waste liquid treatment. Although ionic liquids are not toxic, they are high-cost and highly corrosive too. The efficiency in production will be reduced due to excessive viscosity. Common mechanical preparation methods for CNF include high-pressure homogenization, microfluidic homogenization, the ultrafine grinding method and the ball milling method. These methods usually consume a lot of energy [11]. Ultrasonic treatment is a green preparation method developed in recent years, which has the advantages of being green and having low energy consumption. Chen et al. prepared CNF from wood, bamboo, wheat straw and flax fibers with an ultrasonic power of 1000 W [12]. However, ultrasonic waves also promote aggregation between CNFs [13], and affect the dispersion of CNFs. Research has found that the interaction of cellulose molecules with low molecular weight liquids can greatly affect the physical and chemical processing of cellulose [14]. For example, during mechanical treatment, adding low molecular weight liquids, such as dimethyl sulfoxide (DMSO), can reduce energy consumption [15]. Therefore, this work proposes soaking durian rind cellulose in DMSO before ultrasonic treatment, so as to improve the preparation efficiency of cellulose nanofiber. One of the potential applications of CNF is for aerogel. At present, there have been some reports on aerogels from CNF produced from agricultural and forestry wastes. Fukuda et al. used almond shell as raw material to prepare a CNF aqueous suspension by the TEMPO oxidation method, and then obtained aerogel by the freeze-drying method with porosity as high as 99.9% [16]. Hosseini et al. prepared a CNF suspension from date palm waste and obtained aerogel with heavy metal adsorption capacity by the freeze-drying method [17]. The aerogel had good adsorption performance for chromium (VI), lead (II) and mercury (II) ions.

Here, agricultural and forestry waste durian rind was used as a raw material to explore the preparation of cellulose nanofiber by combining low molecular weight liquid DMSO and the ultrasonic method. The extraction of cellulose from durian rinds and the preparation of cellulose nanofiber were explored. The application of durian rind-based CNF in the aerogel field is also discussed regarding the relationship between microstructure and mechanical properties.

## 2. Results and Discussion

### 2.1. Extraction of Durian Rind-Based Cellulose and Preparation of Cellulose Nanofiber

A green and environmentally friendly method was used to extract cellulose from durian rinds and prepare cellulose nanofiber. For extraction of cellulose from durian rinds, mild sodium carbonate was used to remove lignin and hemicellulose, instead of the popular sodium hydroxide [18,19]. Sodium hydroxide, which is a strong alkali, causes corrosion in equipment and certain risks in operation, as well as difficulty in and economic pressure on wastewater treatment. Sodium carbonate is a more environmentally friendly, economic and safe choice. The extraction yield of cellulose from durian rinds was as high as 32.23% via this technology, which was comparable to the content of cellulose in durian rinds. Hence, it is an efficient and green method.

In the process of preparing cellulose nanofiber, the green method of ultrasonic dispersion was selected. An ultrasonic wave is a mechanical wave with an extremely short wavelength that propagates more easily in liquid media. As the energy of the ultrasonic wave increases, a large number of small bubbles are created in liquid. These small bubbles grow and then burst suddenly. During this process, huge energy is released. Collision probability and intensity between cellulose molecules increase. The intermolecular hydrogen bonds among cellulose molecules are gradually destroyed, and finally cellulose is dissociated into nanofiber. Ultrasonic waves could promote aggregation between CNFs [13], which has an unfavorable effect on dispersion of CNFs. Therefore, we soaked durian rind-based cellulose in DMSO solution before ultrasonic treatment. DMSO is a good hydrogen bond breaker by forming stronger hydrogen bonds with hydroxyl groups of cellulose, which can break hydrogen bonds between cellulose molecules and hinder aggregation. Based on this principle, the power of ultrasonic treatment was successfully reduced to only 360 W to prepare CNF here. After most of the DMSO was removed in the subsequent centrifugation and washing stage, a trace amount of DMSO remained in the CNF suspension, which was beneficial to the stability and preservation of the CNF suspension.

### 2.2. Performance of Durian Rind-Based CNF

**Figure 1:**
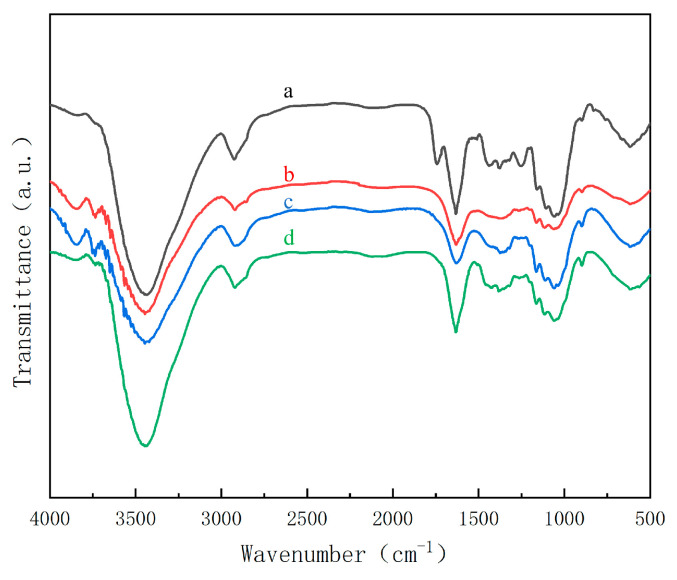
FTIR spectra ((a) Washed durian rind powder; (b) Sample treated with sodium carbonate; (c) Sample treated with sodium chlorite; (d) Sonicated sample).

In order to understand the effect of sodium carbonate treatment on removal of lignin and hemicellulose from durian rinds, as well as the effect of ultrasonic treatment on cellulose, FTIR spectra of samples at each stage were recorded, as shown in Figure 1. The peak at 1742 cm^−1^ was due to the stretching vibration of C=O, an ester group present in hemicellulose. The C=C skeleton vibration at 1512 cm^−1^ indicated the presence of the benzene ring of lignin. The C-H bending vibration in lignin-CH_3_ was at 1438 cm^−1^. The C-O stretching vibration peak of the lignin aromatic base was at 1250 cm^−1^ [20,21]. All these characteristic peaks were presented in FTIR spectra of the washed durian rind powder. After treatment by sodium carbonate, these characteristic peaks disappeared in curve b, indicating that hemicellulose and lignin in durian rinds had been successfully removed. Compared to curve b, curve c did not change significantly, indicating that sodium chlorite only played the role of bleaching and had no effect on the chemical structure of cellulose.

In the FTIR spectrum of cellulose, the characteristic absorption peaks of cellulose include the absorption peaks of the β-glycosidic bond at 895 cm^−1^, C-O stretching vibration at 1055 cm^−1^, C-H bending vibration at 1376 cm^−1^, C-H bending vibration at 2925 cm^−1^ and O-H stretching vibration at 3433 cm^−1^ [22]. These characteristic peaks were present in every curve in Figure 1. This showed that the processes, including the sodium carbonate treatment, sodium chlorite bleaching and ultrasonic dispersion, did not affect the chemical structure of cellulose. Therefore, the method successfully removed lignin and hemicellulose, and effectively preserved cellulose.

**Figure 2:**
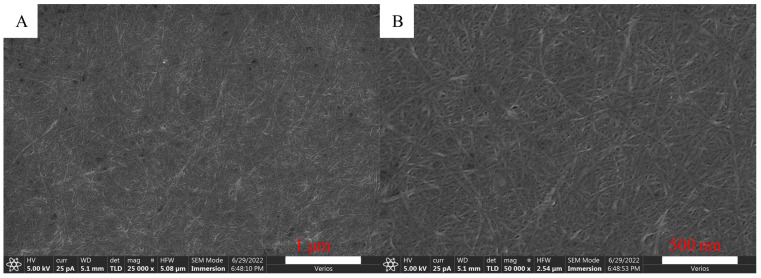
SEM images of CNF ((**A**) Low magnification; (**B**) High magnification).

The SEM images of CNF are shown in Figure 2. CNF suspension was dropped on a silicon wafer and then dried. As water evaporated, CNFs gathered and moved closer to each other under the attraction of hydrogen bonds between water and CNF, and finally densely deposited on the silicon wafer. CNFs were clearly observed from the SEM images. Using Nanomeasurer1.2 to analyze CNF size, the diameter was basically 8–20 nm, and the length was several micrometers.

**Figure 3:**
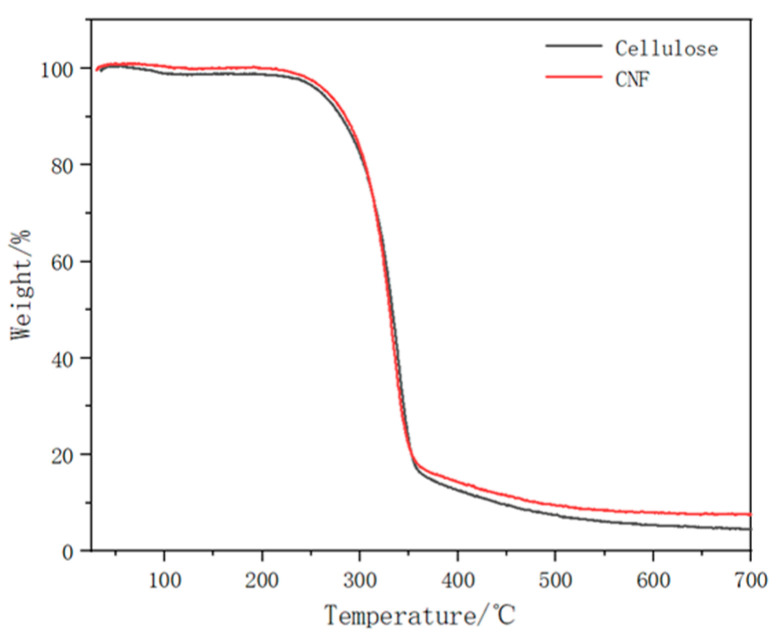
TG curves of cellulose and CNF from durian rinds.

The TG curves of cellulose and CNF from durian rinds are shown in Figure 3. The TG curve of CNF was quite similar to cellulose. Both of them had only one pyrolysis temperature at about 330 °C. It indicated that the products had high purity and stable thermal properties.

**Figure 4:**
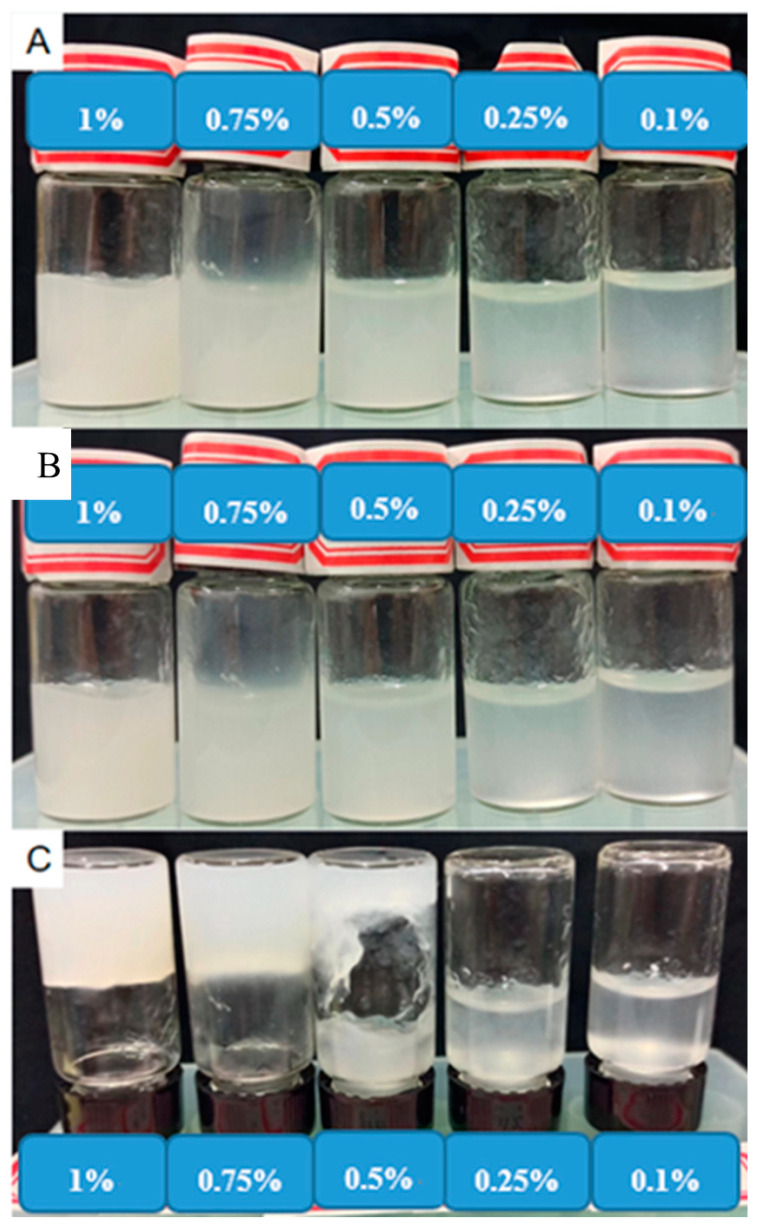
CNF suspensions at different concentrations ((**A**) Day 1; (**B**) Day 30; (**C**) Inverted after 30 days).

CNF suspension was diluted to different concentrations and placed in sample bottles at room temperature for 1 month to evaluate dispersion and stability. It is seen from Figure 4A that the samples of various concentrations were able to disperse uniformly at first. After standing for one month, as shown in Figure 4B, no obvious coagulation phenomenon happened. All samples showed good stability. Inverting the samples as shown in Figure 4C, CNF suspensions with 0.1% and 0.25% concentration still had strong fluidity, while the 0.5% sample had weakened fluidity. Moreover, the 1% sample had no fluidity at all and formed gel. This was attributed to the high aspect ratio of CNF. When the concentration increased to 0.5%, CNFs aggregated with each other under the interaction of hydrogen bonds, and the mobility decreased. As the concentration increased to 1%, a three-dimensional network structure formed, resulting in the loss of fluidity of the CNF suspension and the formation of gel. In addition, the zeta potential of the 0.1% CNF suspension was as low as −63 mV, which also indicated that the CNF suspension had excellent stability and was negatively charged.

### 2.3. Application of Durian Rind-Based CNF in Aerogel

Due to abundant pores, aerogels have many excellent properties. As a new aerogel, cellulose aerogel with both degradability and sustainability has attracted more attention. Although cellulose aerogels prepared by alkaline methods have generally stronger mechanical properties than traditional silica aerogels, they still have not met the practical demand in wider applications. This is because, for cellulose aerogels prepared by the alkaline methods, the cellulose type is usually cellulose II, which is regenerated cellulose. The type of natural cellulose is cellulose I. The difference between these two cellulose types lies in the hydrogen bonding pattern. Cellulose I exhibits a flat orientation of macromolecular chains, while cellulose II exhibits an antiparallel orientation. The elastic modulus of cellulose I is higher than that of cellulose II, so the mechanical properties of aerogel composed of cellulose I are usually better [23]. Moreover, the skeleton structure tends to shrink to a certain extent during the preparation of regenerated cellulose aerogel. The durian rind-based CNF obtained in this work was assigned to cellulose I, which could make a positive influence in cellulose aerogel.

**Figure 5:**
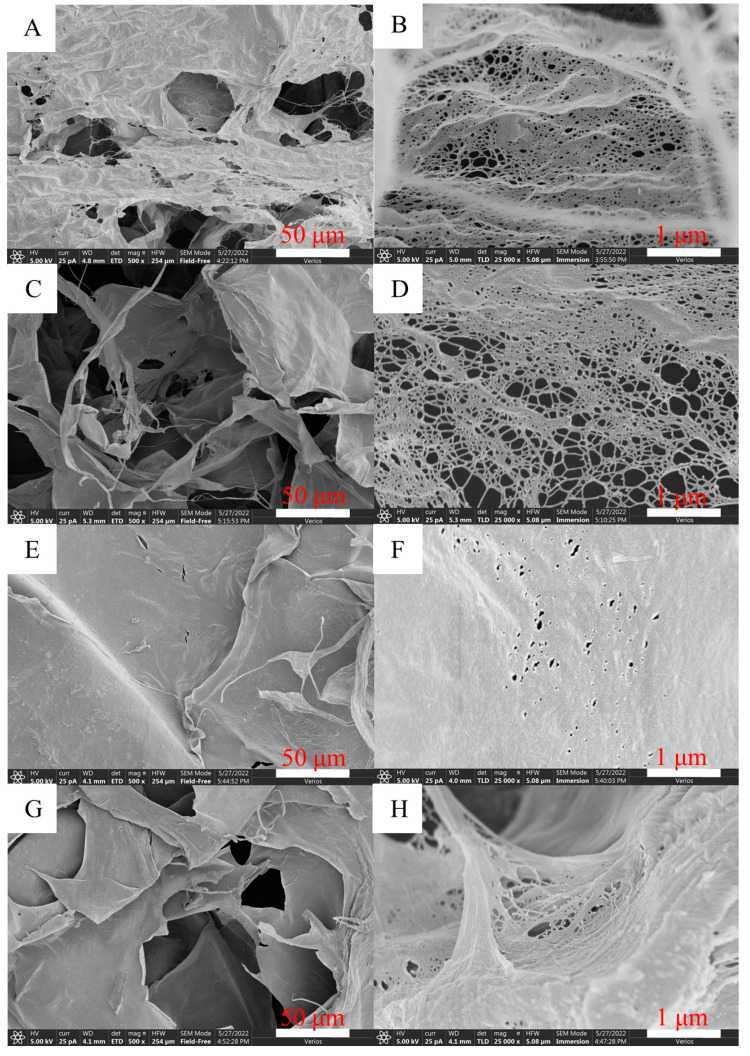
SEM images of CNF aerogels ((**A**,**B**) CNFA0.25; (**C**,**D**) CNFA0.5; (**E**,**F**) CNFA0.75; (**G**,**H**) CNFA1)).

Figure 5 shows microscopic morphologies of pure CNF aerogels from the durian rind-based CNF suspension. During the freezing process of the CNF suspension, water gradually solidified into ice crystals. Under the push of ice crystals, CNFs aggregated and entangled with each other under the interaction of hydrogen bonds to form a network skeleton, which formed aerogel after being freeze-dried. CNF aerogels with different morphologies formed with different concentrations of CNF suspensions. CNFA0.25 (Figure 5A,B) possessed a uniform porous network structure. For CNFA0.5 (Figure 5C,D), due to the increased concentration, the hydrogen bonds between CNFs were enhanced and arrangement was tighter, leading to decreased micropores and the appearance of larger pores. With the further increase in concentration, most of the micropores disappeared in CNFA0.75 (Figure 5E,F), and the sheet structure appeared. With the highest concentration, CNFA1 (Figure 5G,H) exhibited a compact and neat sheet structure, and the supporting fiber structure was observed between layers. The influences of CNF suspension concentration on the microstructure were in accordance with previous studies [24,25].

**Figure 6:**
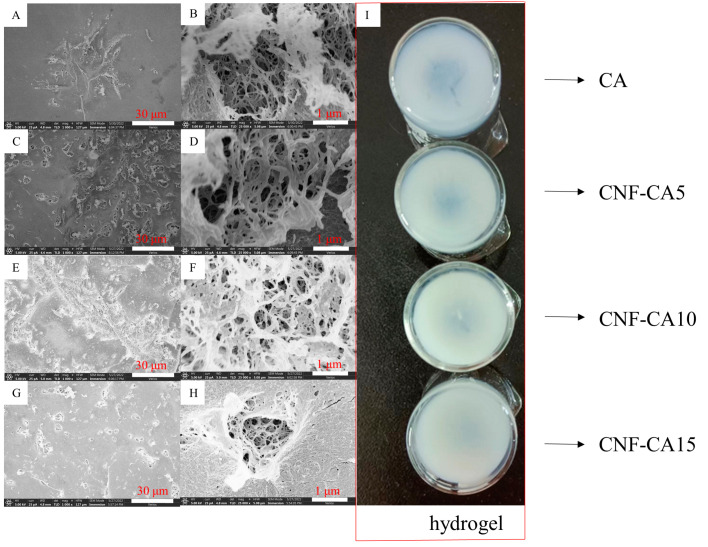
SEM images of composite cellulose aerogels ((**A**,**B**) CA; (**C**,**D**) CNF-CA5; (**E**,**F**) CNF-CA10; (**G**,**H**) CNF-CA15)) and macroscopic pictures of corresponding hydrogels (**I**).

Durian rind-based CNF, as a reinforcing phase in cellulose aerogel, had a significant effect on its microscopic morphology. As shown in Figure 6I, the hydrogel prepared with only cotton linter cellulose showed obvious cracks due to shrinkage during the process of cellulose regeneration. Under the premise that the total amount of cellulose was 3%, with the increase in the replacement amount of durian rind-based CNF, the cracks gradually decreased. The cracks disappeared completely when the replacement amount reached 15%.

In CA SEM images (Figure 6A,B), a tightly packed structure and large voids were observed. Very large cracks appeared. In the composite cellulose aerogels with CNF, cellulose with longer length provided the main skeleton structure. Due to the mechanical treatment, the macromolecular chain was partially broken, leading to the length of molecular chains being reduced. Hence, CNF can fill in the skeleton as a reinforcing phase. For CNF-CA5 (Figure 6C,D), the smaller length and diameter of CNF provided more contact points between cellulose fibers, and made the cellulose skeleton stronger and the shrinkage effect decreased. In the SEM images, a sparser structure was observed and the cracks had decreased. With more CNF, the structure of CNF-CA10 (Figure 6E,F) was sparser, and the cracks reduced further. As the CNF content reached 15%, in CNF-CA15 (Figure 6G,H), CNFs also aggregated with each other so that CNFs were densely packed in the backbone. Therefore, a tightly packed structure was seen and the cracks disappeared completely.

**Figure 7:**
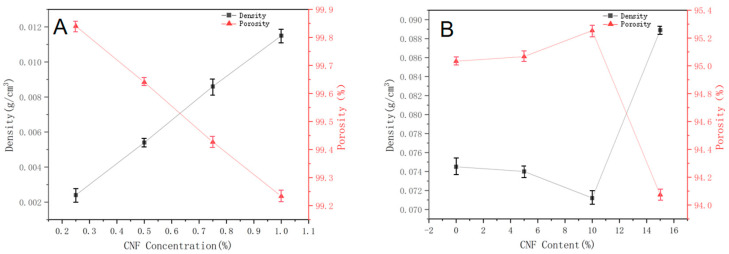
Variations in density and porosity of CNF aerogel (**A**) and the composite cellulose aerogel (**B**).

Figure 7 shows the variation in density and porosity of CNF aerogel and the composite cellulose aerogel. As shown in Figure 7A, the density of CNF aerogel gradually increased from 0.0024 g/cm^3^ to 0.0115 g/cm^3^ with the increase in CNF suspension concentration. This was due to the excellent mechanical properties of cellulose I so that there was no obvious volume shrinkage in the process of freeze-drying. The density of CNF aerogel gradually increased as a linear law with the increase in CNF concentration. At the same time, the porosity gradually decreased from 99.84% to 99.23%. This was because the number of CNFs per unit volume increased and the interaction between CNFs was stronger as the concentration increased. Under the interaction of hydrogen bonds, more CNFs were arranged more tightly, resulting in a decrease in the number of pores. All CNF aerogels had porosity above 99%.

As shown in Figure 7B, the density of the composite cellulose aerogel showed a trend of first decreasing and then increasing with the increase in CNF content. Correspondingly, the porosity first increased and then decreased. With the increase in CNF content from 0 to 10%, CNF was uniformly filled in the cellulose skeleton. The supporting effect of CNF on the aerogel skeleton was obvious so that the shrinkage of the aerogel decreased. Therefore, the density of the composite aerogel gradually decreased from 0.0745 g/cm^3^ to 0.0712 g/cm^3^, and the porosity increased from 95.03% to 95.25%. When the CNF content reached 15%, the aerogel shrank obviously due to less cellulose in the skeleton and the enhanced aggregation of CNFs. At the same time, the density increased to 0.0889 g/cm^3^ and the porosity decreased to 94.0733%. This phenomenon was consistent with the results observed in the SEM images.

**Figure 8:**
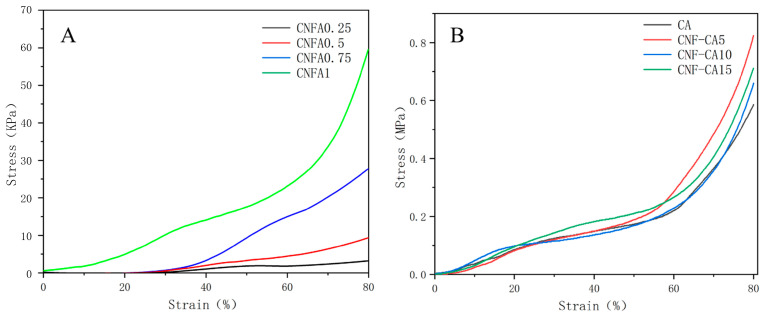
Compressive stress and strain curves of CNF aerogel (**A**) and composite cellulose aerogel (**B**).

In order to explore the reinforcement effect of durian rind-based CNF on cellulose aerogel, the compressive stress–strain curves of CNF aerogel and composite cellulose aerogel were measured, as shown in Figure 8. As shown in Figure 8A, as the concentration of CNF increased, the stress of CNF aerogel increased significantly. The improvement of mechanical properties was closely related to the density and porosity of aerogel. During the whole compression process, the aerogel did not break and had good flexibility. The concentration of CNF had a great influence on the pore structure of aerogel, as discussed for the SEM images. With the increase in concentration, the aerogel gradually changed from a porous network to a densely arranged sheet braced by fibers, which gave the aerogel better compression performance.

As shown in Figure 8B, for the composite cellulose aerogels, the stress values were significantly larger than those of pure CNF aerogels. The reason was that the cellulose concentration in the composite cellulose aerogel was much higher than that in the CNF aerogel. So, cellulose fibers in the composite cellulose aerogel were arranged more densely, resulting in a higher compressive strength. At the same time, it was also observed that the addition of CNF further improved the compression strength of the composite cellulose aerogel. The maximum compressive stress of CA was only 0.586 MPa, which was far lower than the maximum compressive stress of the composite cellulose aerogels. The maximum compressive stress of CNF-CA5 was 0.824 MPa, which was highest among the three composite aerogels. A small amount of CNF could have been uniformly dispersed in the aerogel skeleton so that the stress was well dispersed. However, the maximum compressive stress of CNF-CA10 decreased to 0.66 MPa when the CNF content increased to 10%. In CNF-CA10, the amount of cellulose was too low to support the skeleton structure, while the amount of CNF phase increased. Thus, the whole structure of the aerogel became looser, resulting in a decreasing compressive strength. For CNF-CA15 with 15% CNF, although the amount of cellulose in the skeleton decreased, more aggregated CNF phases formed, which filled in the aerogel skeleton as a more compact structure. Thus, the compressive stress also increased to 0.711 MPa. Therefore, the compressive strength of cellulose aerogel could be increased by adding an appropriate proportion of CNF. This conclusion was in accordance with previous studies [24].

## 3. Materials and Methods

### 3.1. Materials

Durian rinds were obtained from local fruit stores. Cotton linter fiber was purchased from Hubei Chemical Fiber Group Co., Ltd. (Xiangyang, China). Sodium carbonate and sodium hydroxide were purchased from Guangzhou Chemical Reagent Factory (Guangzhou, China). Sodium chlorite was purchased from Aladdin Reagents Co., Ltd. (Shanghai, China). Glacial acetic acid and absolute ethanol were purchased from Xilong Science Co., Ltd. (Shantou, China). Dimethyl sulfoxide (DMSO) and tert-butanol were purchased from MacLean Reagents Co., Ltd. (Shanghai, China). Urea was purchased from Guangdong Guanghua Chemical Factory Co., Ltd. (Guangzhou, China). The purity of sodium chlorite was 80%, and other reagents were analytically pure. Deionized water was made in the laboratory.

### 3.2. Cellulose Extraction and Nanocellulose Fiber Preparation

The durian rinds were pulverized into a fine powder with a pulverizer and washed with ethanol and boiling water. One gram of washed durian rind powder was put into 40 mL of 15 wt.% sodium carbonate solution, stirred at 100 °C for 4 h and then filtered. One gram of powder was washed until pH was neutral and then put into 40 mL of 1.75 wt.% NaClO_2_ solution. pH of the solution was adjusted to 3–4 with glacial acetic acid. The powder was bleached at 75 °C for 30 min, then filtered and washed to neutral. Finally, durian rind-based cellulose was obtained after lyophilization. All washing processes were conducted through adding water and centrifugation with rate 8000 r/min for 5 min.

Four grams of durian rind cellulose was soaked in 32 mL of DMSO solution for 24 h, then placed in 2000 mL deionized water and sonicated at 360 W for 30 min using an ultrasonic cell disruptor with a 4-second pause for each 4-second ultrasonic treatment. After ultrasonic treatment, centrifugation was performed at a speed of 8000 r/min for 5 min. The upper layer solution was CNF suspension, and the precipitate was incompletely separated cellulose. The CNF suspension was centrifuged at 18,000 r/min for 10 min. The final precipitate was washed with water several times to remove DMSO to obtain durian rind-based CNF.

### 3.3. Preparation of Aerogel

The above-mentioned durian rind-based CNF was formulated into suspensions with 0.25%, 0.5%, 0.75% and 1% concentrations, respectively, which were placed in a −18 °C refrigerator for 24 h. The pure CNF aerogels were obtained after lyophilization. According to the concentration, CNF aerogels were named as CNFA0.25, CNFA0.5, CNFA0.75 and CNFA1, respectively.

A certain amount of cotton linter fiber was added into the sodium hydroxide–urea alkali solution with a mass ratio of sodium hydroxide, urea and water of 7:12:81, and the mass fraction of cotton linter fiber was kept at 3%. The mixture was frozen in a refrigerator at −18 °C, and then taken out, thawed and stirred. The process of freezing–stirring and thawing was repeated until cotton linter fiber was completely dissolved and the solution was transparent. Then, the solution was heated up to room temperature. Ethanol was slowly added into the solution to promote gelation. The hydrogel was formed after 24 h. The hydrogel was soaked in deionized water until neutral. Finally, cellulose aerogel (CA) was obtained after lyophilization. Composite aerogels were prepared by the same method with part of the cotton linter fiber replaced with durian rind-based CNF. The replacement amount of CNF was 5%, 10% and 15%, respectively. The corresponding samples were named as CNF-CA5, CNF-CA10 and CNF-CA15, respectively.

### 3.4. CNF Characterization

A Fourier transform infrared spectrometer (TENSOR27, Bruker, Germany) was used for characterizing samples’ chemical structure with wavenumber 400–4000 cm^−1^. Samples were prepared by a pellet method. After being fully dried, samples were evenly mixed and ground with KBr. After being pelleted, samples were placed in the infrared spectrometer to test infrared spectra.

In order to understand microscopic morphology of CNF, a CNF suspension with 0.1% concentration was dropped on a silicon wafer, and dried in an oven. The sample was sprayed with gold and observed by a field emission scanning electron microscope (Verios G4 UC, Thermo Fisher Scientific Brno Ltd., Waltham, MA, USA).

In order to explore thermal stability of products, thermogravimetric curves were measured by a comprehensive thermal analyzer (ZCT-1, Beijing Jingyi Hi Tech Instrument Co., Ltd., Beijing, China). The durian rind-based cellulose and CNF were fully dried in an oven beforehand. The heating rate was set to 10 °C/min and the temperature range was set from 25 °C to 700 °C. The whole process was carried out in nitrogen atmosphere.

To evaluate stability, the CNF suspension was diluted to 0.1%. The zeta potential was measured with a nanoparticle size and zeta potentiostat (Zetasizer, Shanghai Yuzhong Technology Co., Ltd., Shanghai, China). At the same time, CNF suspensions with concentrations of 1%, 0.75%, 0.5%, 0.25% and 0.1%, respectively, were left for 1 month to observe stability.

### 3.5. Aerogel Characterization

Micromorphology of aerogels was observed using a field emission scanning electron microscope (Verios G4 UC, Thermo Fisher Scientific Brno Ltd., Waltham, MA, USA). Aerogels were sprayed with gold, and then put into the instrument for observation.

Samples were dried to constant weight at 60 °C in a blast air-drying oven, and then mass (*m*_0_) was weighed with an electronic balance. Height and diameter of samples were measured with a vernier caliper, and volume (*V*) was calculated. Density (*ρ*) of aerogels was calculated according to Equation (1).

(1) $$\rho =\frac{m_{0}}{V}$$

Porosity (P) of aerogels was calculated using Equation (2).

(2) $$P=(1-\frac{\rho_{m}}{\rho_{t}})\times 100\%$$

*ρ_m_* is the volume density of aerogel. *ρ_t_* is the skeleton density of cellulose, which is 1.5 g/cm^3^.

The compressive properties of aerogels were tested with a universal testing machine (WDW-5, Jinan Hengxu Testing Machine Technology Co., Ltd., Jinan, China). The compressive stress–strain curves were obtained with the compression rate 2 mm/min and strain of 80%.

## 4. Conclusions

In this work, we successfully extracted cellulose and prepared cellulose nanofiber from agricultural and forestry waste durian rinds via a green, highly efficient and low energy consumption method aided by the combination of low molecular weight liquid DMSO and the ultrasonic method. The power of ultrasonic treatment was low to only 360 W. The diameter of durian rind-based CNF was 8–20 nm, and the length was at the micrometer scale. The durian rind-based CNF suspension had good dispersibility and stability in water. The porosity of pure CNF aerogel was high to 99%. The concentration of CNF had a great influence on the microstructure and mechanical properties of CNF aerogels. CNF aerogel with 1% CNF showed a sheet structure with supporting fibers, as well as the strongest compression performance. CNF had an obvious reinforcing effect on the composite cellulose aerogel. The composite cellulose aerogel with 5% CNF has the highest compressive strength. These achievements provide a beneficial scientific reference for durian rind-based CNF’s application in the field of aerogels. Durian rind-based CNF is expected to have more potential applications in material storage, water treatment, sound insulation, cosmetics, food and coating fields.

## Data Availability

The data presented in this study are available on request from the corresponding author.
